# A Robust Control Group Identification Approach for Reference Intervals of Thyroid Biomarkers: The Case of Jordan

**DOI:** 10.3390/medsci14030362

**Published:** 2026-06-30

**Authors:** Areej Mohammed, Hussam Alshraideh, Munir Abu-Helalah, Abdulrahim Shamayleh

**Affiliations:** 1 Department of Industrial Engineering, American University of Sharjah, Sharjah P.O. Box 26666, United Arab Emirates; g00092380@alumni.aus.edu (A.M.); ashamayleh@aus.edu (A.S.); 2 Advanced Biosciences and Bioengineering Research Center, American University of Sharjah, Sharjah P.O. Box 26666, United Arab Emirates; 3 Industrial Engineering Department, Jordan University of Science and Technology, Irbid P.O. Box 22110, Jordan; 4 Department of Family and Community Medicine, School of Medicine, University of Jordan, Amman P.O. Box 11942, Jordan; m.abu-helalah@ju.edu.jo

**Keywords:** thyroid-stimulating hormone, free triiodothyronine, free thyroxine, hypothyroidism, hyperthyroidism, reference range

## Abstract

**Purpose:** This study introduces a robust approach for establishing reference intervals (RIs) for thyroid biomarkers in the Jordanian population by incorporating symptoms-based criteria into the control group selection. This method enhances the traditional inclusion–exclusion criteria by adding a symptom layer. Five thyroid biomarkers were analyzed: thyroid-stimulating hormone (TSH), free triiodothyronine (FT3), free thyroxine (FT4), thyroid receptor antibodies (TRAbs), and antithyroid peroxidase antibodies (TPOAb). The study also investigated the prevalence of thyroid disorders using the established population-specific RIs. **Patients and Methods:** Samples from 6782 participants across different regions were collected between June 2016 and May 2017. The Kruskal–Wallis and the Mann–Whitney tests identified the best partitioning based on age–gender significant differences, and 2.5th–97.5th percentiles were used to establish the RIs. **Results:** The TSH, FT4, and FT3 differed significantly among males and females, while no significant differences were found among different age groups. The RIs for males were as follows: TSH, 0.50–4.18 mIU/L; FT4, 12.00–21.16 pmol/L; and FT3, 2.28–4.17 pmol/L, while the corresponding RIs for females were as follows: TSH, 0.57–4.61 mIU/L; FT4, 12.02–24.69 pmol/L; and FT3, 2.53–3.71 pmol/L. TRAbs and TPOAb RIs were 0.3–1.89 IU/L and 0.83–23.87 IU/mL, respectively. The prevalence of overt hypothyroidism was 10.63%, subclinical hypothyroidism was 3.53%, overt hyperthyroidism was 0.66%, and subclinical hyperthyroidism was 1.52%. **Conclusions:** This study emphasizes the role of population-specific RIs in minimizing diagnostic errors. The addition of symptom criteria improved control group identification and RI accuracy. These findings improve the diagnosis of thyroid disorders in Jordan and suggest a reproducible framework for worldwide application.

## 1. Introduction

The levels of thyroid hormones determine appropriate growth and the optimal functioning of physiological systems [1]. An assessment of the prevalence of thyroid disorders and the factors that influence them, such as age, gender, and ethnicity, is necessary to understand the mechanisms underlying variations in thyroid hormone levels. These disorders are assessed using different biomarkers, including thyroid-stimulating hormone (TSH), free triiodothyronine (FT3), and free thyroxine (FT4) [2]. Moreover, high levels of thyroid receptor antibodies (TRAbs) and antithyroid peroxidase (ATPOb) antibodies may indicate signs of autoimmune thyroid disorders [3,4].

In 2017, the American Thyroid Association (ATA) suggested that more precise diagnosis of thyroid diseases could be achieved if each population had its own reference intervals (RI) [5]. Like most nations in the Middle East, the Hashemite Kingdom of Jordan still relies on RIs provided by equipment manufacturers and on data from other countries that do not account for possible gender, environmental, and ethnic differences that could affect thyroid hormone levels, leading to diagnostic inaccuracies. Several studies have revealed that the RIs of thyroid hormones vary with age, sex, and ethnicity, calling for the construction of population-specific RIs for better diagnosis and treatment of diseases [6,7,8,9]. Based on the recommendations of the ATA, Clinical Laboratory Standards Institute (CLSI) [10], and some relevant literature [6,7,8,9]. This study assumes that the general population RIs are inconsistent with those applied to the Jordanian population.

Indirect methods for establishing RIs depend on the data collected by different laboratories; therefore, some limitations do exist [11]. Conversely, direct methods are recommended by CLSI [10] as they are well known to provide a high internal validity and minimal bias. Thus, the current study aimed to establish thyroid hormone RIs for the Jordanian population using a robust method for identifying control groups and considering age–gender differences, employing the direct method. Subsequently, the RIs obtained in the current study will be compared with those previously established in other countries and with those provided in the manufacturer’s instructions. Lastly, the established RIs of the Jordanian population are employed to determine the prevalence of thyroid diseases in Jordan.

## 2. Methods

### 2.1. Materials and Study Design

This study utilizes data from a broader project called “Jordan’s National Hypothyroidism and Hyperthyroidism”, focusing on thyroid disorder prevalence, disease control, symptoms, medication adherence, and quality of life across gender–age groups in Jordan [12]. A cross-sectional study was conducted from June 2016 to May 2017 on adults aged 18–79 from Central, Southern, and Northern Jordan. The sample included two groups: 539 diagnosed with thyroid disorders and 6190 (study group) unaware of their thyroid status. Blood samples from the latter group were analyzed for thyroid hormones, along with a comprehensive assessment of symptoms, chronic diseases, family history, and medications. More details on the sampling process are provided by Abu-Helalah and others [12,13].

Ethical approval for the study was submitted to the Research Ethics Committee at Mutah University in accordance with standard procedures. Ethical research approval was granted by the committee under IRB approval number 201543 dated 4 March 2016. Additionally, necessary approvals were secured from the Directorate of Health in Amman, Irbid, and Karak governorates where the study was conducted. The study was funded by the National Research Office of the Jordanian Ministry of Higher Education. Eligible subjects were interviewed at the healthcare centers in the study areas. Research assistants explained the study protocol and obtained written consent from participants to take part in the study, to review their medical profiles, and to inform their family physicians of their participation. This study was conducted in accordance with the Declaration of Helsinki and local regulations.

In the first phase, RIs for five thyroid parameters, TSH, FT4, FT3, TPOAb, and TRAbs, were developed from 528 control group participants who were identified using comprehensive inclusion and exclusion criteria. This identification process begins with incorporating the traditional exclusion criteria as per the National Academy of Clinical Biochemistry (NACB) guidelines, which exclude participants with high thyroid autoantibodies, personal or family history of thyroid disorders, and medications that affect thyroid gland function. On top of these criteria, another layer was added to the exclusion criteria by considering the symptoms factor. Including symptom-based criteria in control group selection, which was often neglected in the literature, ensures accuracy and reliability. In addition, Symptoms were statistically analyzed to assess their contribution to non-normal thyroid status and their impact on TSH levels. In the second phase, data from 6190 participants were analyzed for thyroid disorders to achieve a more accurate evaluation than manufacturer RIs. Individuals with communication difficulties or temporary residency were not involved in the study.

Chemiluminescent immunoassays (Advia Centaur; Bayer Diagnostics, Newbury, UK) were used to measure serum TSH, FT4, and FT3. The laboratory RI for TSH is 0.4–4.5 mU/L, and the interassay coefficient of variation ranges from 4.4% to 10.9% across the measurement range of 0.41–24.5 mU/L. The assay was calibrated against the second International Reference Preparation 80/558, with a lower reporting limit for TSH at 0.1 mU/L and a manufacturer-quoted mean functional sensitivity of 0.019 mU/L. For FT4, the laboratory reference range is 9.0–20.0 pmol/L, and the interassay coefficient of variation ranges from 8.2% to 9.8% across the measurement range of 8.2–54.9 pmol/L. The reference range for FT3 is 3.5–6.5 pmol/L, with an interassay coefficient of variation ranging from 4.2% to 6.9% across the 4.0–16.0 pmol/L range. The electrochemiluminescence immunoassay (ECLIA) method (Roche) was used to measure anti-thyroid peroxidase antibodies (TPOAb), and the reference range is 0–34 IU/mL. Thyroid receptor antibodies (TRAbs) serum levels were assessed using an Elecsys and Cobase immunoassay analyzer (ECLIA). The TRAbs’ functional sensitivity is between 0.1 and 0.3 lU/L, and the manufacturer-provided RI is 0–0.9 IU/L. All five parameters were measured in the control group participants. The specific biomarker criteria used in diagnosing thyroid dysfunction include subclinical hypothyroidism, diagnosed by elevated TSH with normal FT4 and FT3 levels, and overt hypothyroidism, characterized by elevated TSH and low FT4 or FT3 levels. On the other hand, subclinical hyperthyroidism refers to low TSH with normal FT3 and FT4 and overt hyperthyroidism to low TSH but increased FT3 or FT4 [14]. TSH was measured for all participants. FT4 concentrations were determined in individuals with serum TSH that were not within the normal range according to the manufacturer’s RIs. Serum FT3 was also measured when FT4 was within the normal range [12,14].

### 2.2. Statistical Analysis

The dataset was pre-processed to address missing values and duplicates, followed by statistical analysis to examine the impact of symptoms on the upper and lower bounds of TSH RIs. Data on 32 thyroid-related symptoms were collected for each participant and compared between a healthy control group, selected using careful criteria, and individuals with hypothyroidism or hyperthyroidism based on manufacturers’ RIs. Differences in symptom prevalence between these groups were utilized to calculate weights, which were subsequently used to assign symptom scores for each participant. The equations used for calculating weights and symptom scores are detailed below:

$W_{i}=S_{i}-H_{i}$, where $S_{i}$ represents the prevalence of symptom *i* in the group diagnosed with thyroid-related disorders, namely hypothyroidism and hyperthyroidism, and $H_{i}$ is the prevalence of symptom i in the carefully selected healthy group.

Symptoms score for participant j = $\sum_{i=1}^{32}W_{i}\times r_{i}$, where $r_{i}=1$ if a symptom occurs and $r_{i}=0$ if a symptom is not present.

**Figure 1:**
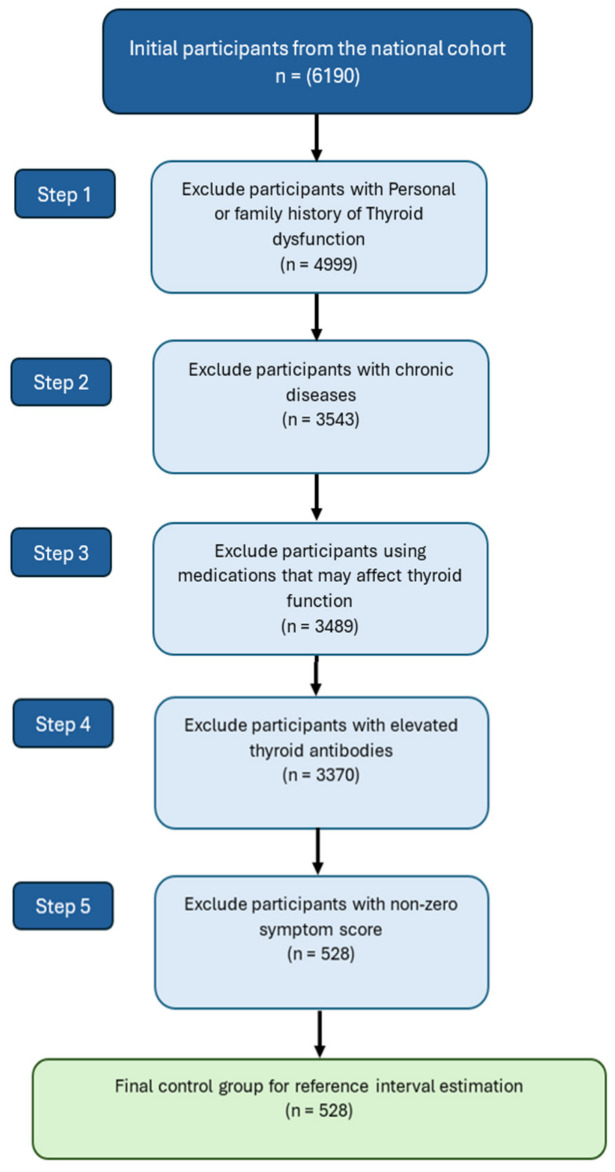
Control group selection flowchart.

Following this, RIs for TSH values were calculated and plotted at various levels of the symptom scores; results were utilized to understand the impact of symptoms on the TSH RIs. Next, to identify the control group, the exclusion criteria outlined by the NACB guidelines were applied. These criteria excluded individuals with a family or personal history of thyroid disorders, elevated antibody levels, chronic diseases, or current medications known to affect thyroid hormone levels, such as levothyroxine, antithyroid drugs, insulin, and steroid hormones. Following this, an additional filtration step was implemented, limiting the group to individuals with a symptom score of zero. A detailed flowchart illustrating the sequential participant exclusion process and the number of individuals excluded at each stage of the control group selection procedure is presented in Figure 1.

Although thyroid-related symptoms may be nonspecific and can occur in healthy individuals, the symptom-based criterion was incorporated as an additional filtration layer to minimize the risk of including participants with undiagnosed thyroid dysfunction who may still meet conventional biochemical inclusion criteria. Requiring a symptom score of zero was intentionally applied to improve the specificity of the healthy reference population during RI construction. According to the NACB and CLSI recommendations, 120 disease-free individuals are sufficient for reliable nonparametric reference interval estimation within each partition. The additional exclusion layer was therefore implemented to increase confidence that the final shortlisted participants represented a truly disease-free population. The symptom-based criterion was not used as a standalone diagnostic tool but rather as an additional conservative filtration step alongside established biochemical and clinical exclusion criteria. Although symptom weighting was initially derived using manufacturer reference intervals, the primary purpose of the symptom-based filtering was to reduce the probability of including individuals with potential thyroid dysfunction within the control group rather than to define thyroid disease status independently. Nevertheless, the possibility of partial incorporation bias cannot be completely excluded and should be considered when interpreting the findings. Furthermore, although some excluded individuals may have been healthy, the final control group still exceeded the recommended minimum sample size required for RI construction, which implies that the final control group is representative as it includes participants from multiple regions across Jordan with the maximum probability of being healthy, maintains sufficient population diversity, and satisfies the minimum sample size requirement to support reliable reference interval construction.

Q-Q plots for the control group data assessed parameter distributions (TSH, FT4, FT3, TPOAb, and TRAbs) within the control group, but non-normality persisted. Subsequently, Mann–Whitney and Kruskal–Wallis tests were applied for age–gender significance analysis. Population-specific RIs were then constructed using 2.5th and 97.5th percentiles, covering 95% of the population [10]. The final control group included 245 males and 283 females, exceeding the CLSI-recommended minimum sample size of 120 disease-free individuals per partition required for reliable nonparametric reference interval estimation. The lower (2.5th percentile) and upper (97.5th percentile) reference limits were estimated using the nonparametric percentile method, and 90% confidence intervals were calculated for both limits. According to CLSI recommendations, potential outliers in reference interval studies should be carefully evaluated using graphical inspection and clinical or analytical judgment rather than being automatically excluded using statistical methods alone. CLSI further recommends that, unless extreme observations are clearly identified as aberrant values resulting from analytical, procedural, or preanalytical errors, emphasis should generally be placed on retaining rather than deleting such observations [10]. Therefore, in the present study, potential outliers were assessed through visual inspection of histograms and boxplots, combined with careful evaluation for any clearly aberrant analytical or procedural observations. Furthermore, the implementation of the strict NACB exclusion criteria, along with the symptom-based filtration layer during the control group selection process likely eliminated most extreme observations prior to RI construction. The selected control group was then reassessed to identify any remaining potential outliers. Nevertheless, since no clear analytical or procedural errors were identified during this second assessment step, the remaining points were considered biologically plausible and retained as per CLSI recommendations.

**Figure 2:**
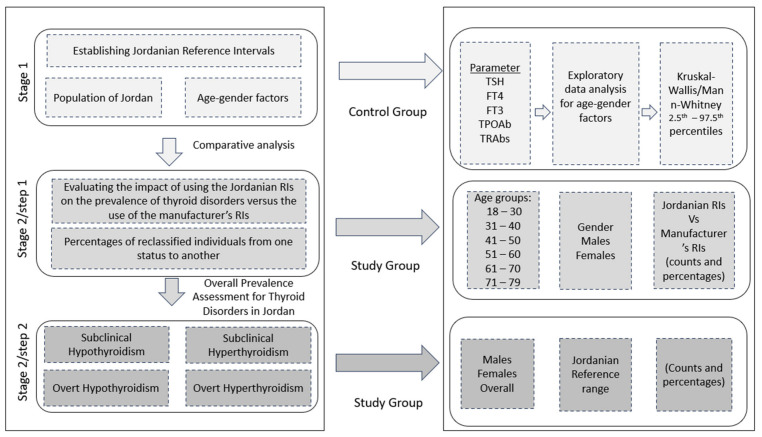
Data analysis framework.

In the second phase, the study group was analyzed to compare the use of Jordanian-specific RIs against manufacturers’ RIs and reclassify thyroid disorder diagnoses. Following that, the overall prevalence of thyroid disorders was calculated by combining the percentage of individuals with known thyroid disorders found in [12] and the percentages for thyroid disease diagnosis for the group with unknown thyroid status using the Jordanian RIs constructed in our research. The prevalence estimates therefore reflected the combined burden of previously diagnosed thyroid disorders, regardless of treatment status, and newly identified biochemical dysfunction cases detected using the Jordanian-specific RIs. The data analysis procedures for the study’s first and second phases are illustrated in Figure 2. The statistical analysis used Python version 3.12 packages, including Numpy version 2.5.0, Pandas version 3.0.3, Matplotlib version 3.11.0, Seaborn version 0.13.2, and Scipy version 1.18.0 [15].

## 3. Results

**Figure 3:**
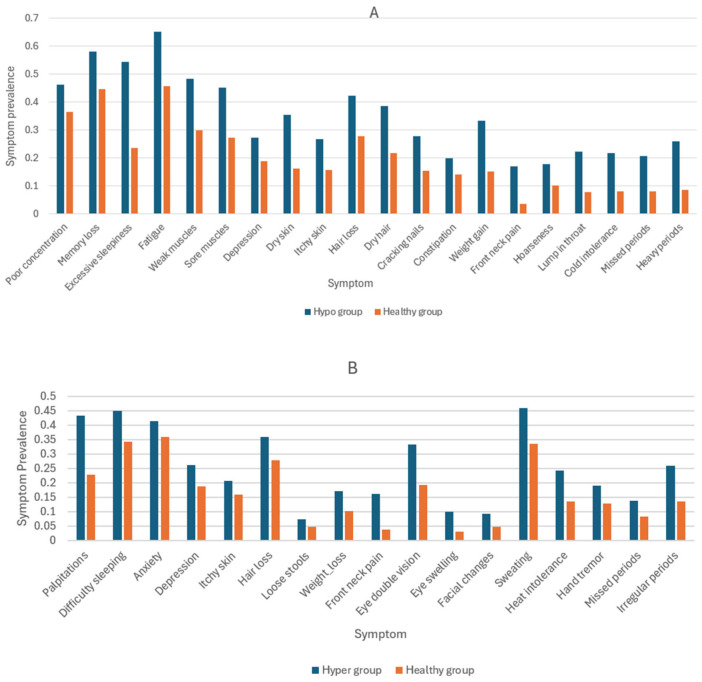
(**A**) Hypothyroidism symptom prevalence in healthy vs. hypothyroid individuals; (**B**) hyperthyroidism symptom prevalence in healthy vs. hyperthyroid individuals.

The prevalence of thyroid symptoms is illustrated in Figure 3; part (A) shows bar graphs for the prevalence of thyroid-related symptoms for the healthy group vs. hypo group, while part (B) shows bar graphs for the prevalence of thyroid-related symptoms for the healthy group vs. hyper group. Clearly, higher prevalence is evident in the sick group compared to the healthy group. Fatigue, memory loss, and poor concentration were the most common symptoms in thyroid patients, aligning with the results of Abu-Helalah and others [16]. Conversely, the least common symptoms were swelling at the front of the neck, protrusion of the eyes, and changes in facial appearance. All 32 symptoms were found to have positive weights, indicating that none of the symptoms had a higher prevalence among healthy individuals compared to those with thyroid disorders. These positive weights confirm that each symptom contributes to identifying a non-normal thyroid status and validate their relevance in distinguishing thyroid dysfunction.

**Figure 4:**
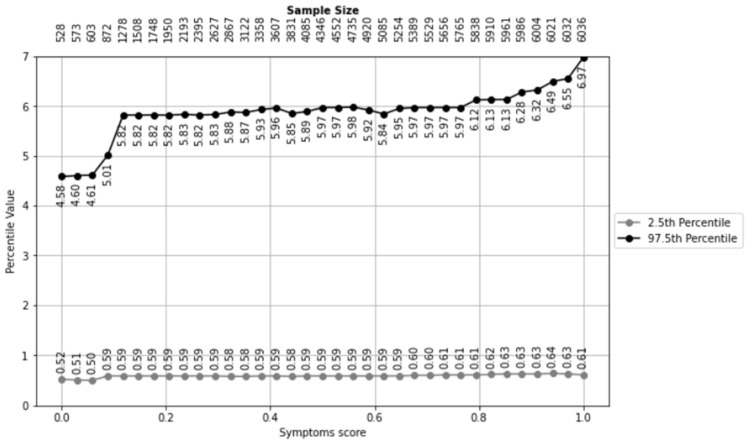
Change in 2.5th and 97.5th percentiles based on the utilized symptoms’ score values.

Figure 4 shows the 2.5th and 97.5th percentiles of TSH at different levels of the symptoms score. At each threshold value, participants with symptoms scores lower than the threshold value used were selected, and their 2.5th and 97.5th percentiles were calculated. A significant increase in upper TSH RI was observed among individuals with symptom scores above 0.75. Besides considering the exclusion criteria mentioned earlier, participants with a symptom score of 0 were used to establish the control group, consisting of 528 individuals.

**Figure 5:**
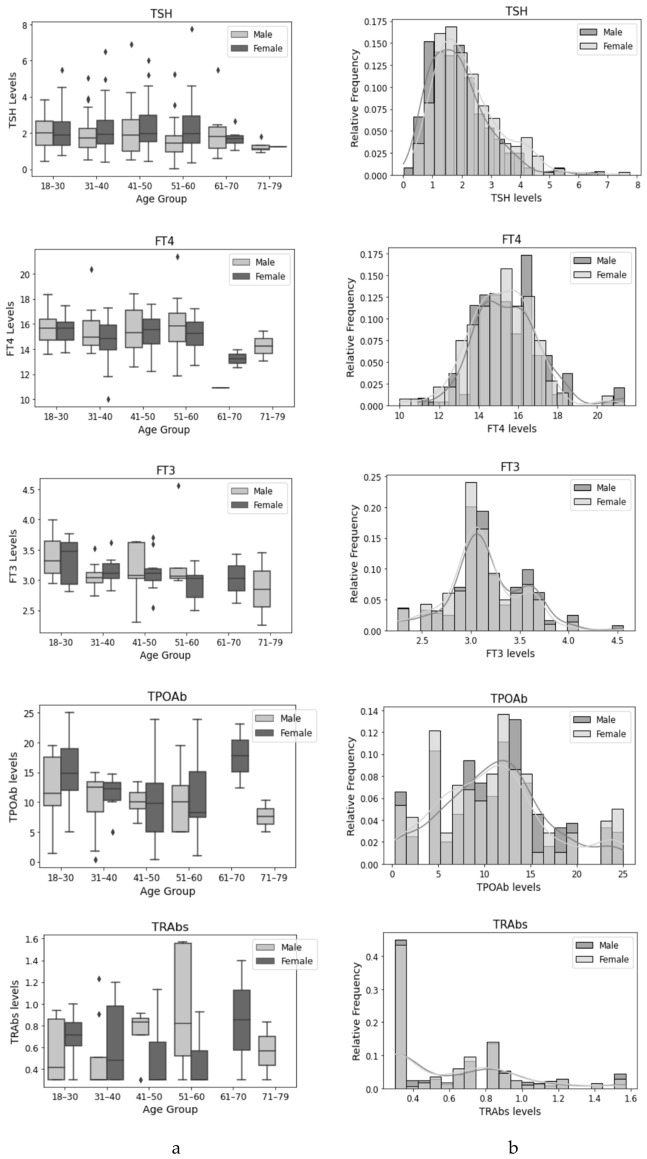
(**a**) Distributions of TSH, FT4, FT3, TPOAb, and TRAbs by age group and gender. Boxes represent the interquartile range (IQR), the central line indicates the median, whiskers extend to 1.5 × IQR, and rhombus markers indicate potential outliers. (**b**) Relative frequency distributions of TSH, FT4, FT3, TPOAb, and TRAbs by gender.

Q-Q plots revealed non-normality for thyroid parameters distribution (TSH, FT4, FT3, TPOAb, and TRAbs), leading to a non-parametric approach. Next, an examination of the gender factor was conducted for each biomarker, as demonstrated in Figure 5b; the serum levels of TSH, TRAbs, and FT3 for males and females display right-skewed distributions. Females exhibited higher median values for TSH, FT3, and TPOAb (1.91 mlU/L, 3.11 pmol/L, 12.07 IU/mL, respectively) compared to males (1.72 mlU/L, 3.07 pmol/L, 11.37 IU/mL, respectively). In contrast, for FT4 and TRAbs, median values for females (15.30 pmol/L, 0.48 IU/mL, respectively) were lower than those for males (15.32 pmol/L, 0.52 IU/mL, respectively). Man–Whitney test revealed significant differences in TSH, FT4, and FT3 (*p* = 0.0014, 0.021, 0.047) but not for TPOAb and TRAbs. Consequently, RIs for TSH, FT4, and FT3 were partitioned by gender, while TPOAb and TRAbs were not. The age values were divided into six consecutive 10-year intervals, from 18 to 80 years, consistent with recent studies on thyroid RIs [9,17,18]. Box plots were then generated, as depicted in Figure 5a, for the categorized age groups to show age–gender-related variations. As illustrated in Figure 5a, the TSH median values fluctuate for males with no discernible pattern of consistent change across age groups, where the lowest values are observed in ages 51–60. Females showed almost similar medians for TSH. Disparities in medians between males and females were evident for participants older than 50. Nevertheless, the Kruskal–Wallis test indicated no significant differences among age groups for the five biomarkers (*p* > 0.09). Thus, no age-based partitioning was done when constructing the RIs for the five parameters. Table 1 details the age and gender percentages in the control group, while Table 2 shows the manufacturer’s RIs, along with the 90% confidence intervals for each limit and the sample size for each partition, as well as the obtained Jordanian population RIs for the five biomarkers.

**Figure 6:**
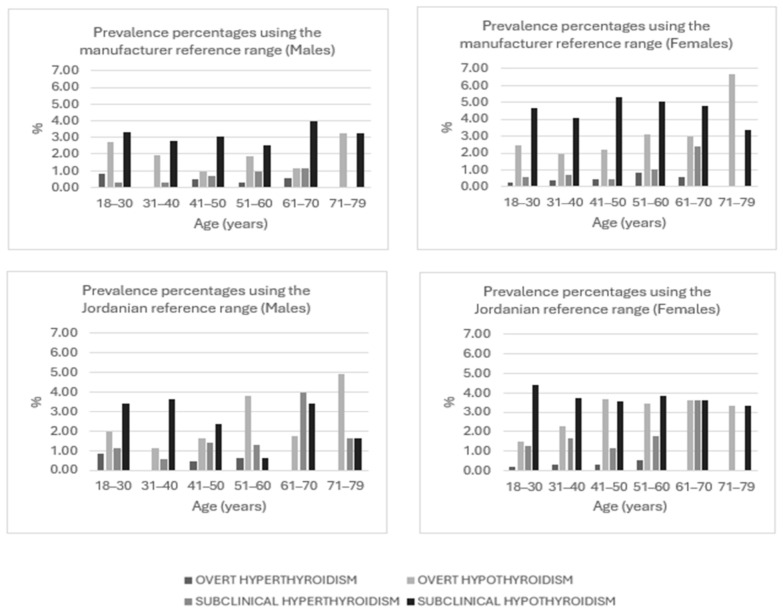
Comparison between the diagnosis of thyroid disorders using the Jordanian RIs versus the manufacturer RIs in males and females.

Table 3 and Table 4 and Figure 6 present thyroid dysfunction diagnoses using manufacturers’ and Jordanian RIs for males and females across age groups, highlighting age–gender variations. In females, the newly established RIs slightly increased overt hypothyroidism (OHypo) and subclinical hyperthyroidism (SCHyper), while overt hyperthyroidism (OHyper) and subclinical hypothyroidism (SCHypo) decreased. The most significant decrease was for OHypo in females over 70 (6.67% to 3.33%), indicating overdiagnosis. In males, OHypo decreased under 40 years but increased above 40. SCHyper increased across all ages with Jordanian RIs, while SCHypo decreased except for males aged 18–30. The diagnosis rates for both genders combined were OHypo (2.45%), SCHypo (3.53%), OHyper (0.33%), and SCHyper (1.52%).

A comprehensive examination of reclassifications using the Jordanian RIs instead of the manufacturers’ Ris was conducted. The percentage of patients reclassified to normal was 0.16%, compared to 1.41% who were reclassified as thyroid patients. Additionally, 1.05% of the participants were reclassified from OHypo to SCHypo compared to 1.41% from SCHypo to OHypo. Reclassification percentages for hyperthyroidism were observed, with only 0.02% of the participants reclassified from SCHyper to OHyper compared to 0.14% reclassified from OHyper to SCHyper. Details about reclassifications are presented in Table 5. Based on the above results, it is evident that the use of the manufacturer’s RIs leads to a significant rate of inaccurate diagnoses. This highlights the critical importance of adopting the RIs specifically for the Jordanian population. The overall prevalence for OHypo and OHyper was calculated by combining the percentage of individuals who have developed thyroid disorder with the ratios derived from the diagnosis of thyroid disease among individuals with an unknown history of thyroid dysfunction. This calculation utilized the Jordanian reference intervals established in our study. According to the study by Abu-Helalah and others [12], which used the same dataset, 539 patients were identified with known hypothyroidism or hyperthyroidism. In total, there were 516 patients with known OHypo (456 females; 57 males) and 23 patients with known overt hyperthyroidism (19 females; 4 males). For prevalence estimation purposes, these previously diagnosed individuals, regardless of treatment status, were combined with the newly classified cases identified in the present study. Upon merging these two groups, individuals with known diseases and those assessed in our study, the overall prevalence of overt thyroid disorders is as follows: OHypo at 10.63% (12.98% for females; 4.99% for males) and OHyper at 0.66% (0.69% for females; 0.59% for males).

## 4. Discussion

This paper introduced a robust approach by incorporating symptoms into the control group’s inclusion–exclusion criteria, distinguishing our work from previous studies that overlooked symptoms during control group selection. This ensures that the control group consists of healthy individuals, reducing the risk of including potential patients with thyroid disorders and enhancing the accuracy and reliability of the established RIs. Our study found that age did not significantly influence the RIs, but gender differed significantly for TSH, FT3, and FT4 levels. These results align with the manufacturer’s reference intervals regarding the non-significance of age as a factor but disagree with applying the same reference intervals for both genders. Additionally, the new Jordanian RIs differ from the manufacturers’, reflecting the impact of ethnicity. Jordanian RIs were higher than the manufacturer’s RIs for all biomarkers except the upper limit for TSH in males and both FT3 limits. The relatively lower FT3 reference intervals observed in the present study compared with the manufacturer’s RIs may reflect population-specific variability, including ethnicity, geographic location, iodine status, nutrition, lifestyle, analytical assay characteristics and the possible effect of the restrictive symptom-based filtering strategy. Given that the study was conducted during a period of improved iodine sufficiency in Jordan following the implementation of the national universal salt iodization program, iodine-related changes in thyroid hormone regulation may have partially contributed to the observed FT3 distributions [19]. Previous studies have also demonstrated that excessive iodine exposure may influence thyroid hormone synthesis through autoregulatory mechanisms such as the acute Wolff–Chaikoff effect. Specifically, high iodine exposure has been associated with reduced organification of iodine and decreased T3 and T4 formation under certain conditions, potentially contributing to variability in thyroid hormone distributions [20]. However, the present study did not directly assess iodine exposure or urinary iodine concentrations at the individual level. Therefore, the possible contribution of iodine-related regulatory mechanisms to the observed FT3 RIs should be interpreted cautiously. In addition, the additional symptom-based filtration layer applied during control group selection may have contributed to the identification of a highly specific disease-free reference population, potentially influencing the observed FT3 interval limits. Although this approach was intentionally implemented to minimize the probability of including individuals with potential undiagnosed thyroid dysfunction, the possibility that the restrictive filtering strategy contributed to the relatively lower FT3 distributions cannot be completely excluded.

Using Jordanian RIs reduced diagnostic inaccuracies, especially for females over 70 who were over-diagnosed using the manufacturer’s RIs. Our study results confirmed the findings in a previous crossover clinical trial by Abu-Helalah and others [16], which enhances the validity of this study’s results. This clinical trial revealed that patients with TSH levels above 4.5 mU/L would benefit from thyroxine replacement, and this cut-off point is more sensitive to clinical response when compared with 4 mU/L or higher levels, such as 5 mU/L. A TSH level of 4.5 mU/L was able to identify patients with clinical improvement when comparing patients with themselves through the cross-over design.

Age–gender trends were explored for five thyroid parameters before constructing RIs, unlike studies that neglected these factors [17,21,22]. However, our findings showed no significant changes in biomarkers with age, contrasting with Yamada and others [9], who reported increased TSH levels with age in the Japanese population. While FT4 levels in females were consistent with those reported by Yamada and others, there was a notable decrease in FT4 for males after age 60, differing from their gradual decline across all ages. Additionally, FT3 levels were higher in males, which is consistent with our results. Tiancheng Xie and others [23] found no significant age-related changes and recommended gender partitioning for FT3. However, their study only reported RIs and did not assess thyroid disorder prevalence using the new RIs.

In the Middle East, the first study on thyroid hormone RIs for Arabs (2006) suggested RIs for TSH (0.30–4.32 mU/L) and FT4 (9.8–18.6 pmol/L) [24], which are lower than the Jordanian RIs. Later studies in Lebanon, Sudan, Iran, and Turkey also aimed to develop population-specific RIs for thyroid hormones [21,25,26]. Comparing these with our findings reveals both similarities and differences. For example, in Lebanon, the lower limit of TSH increased to 0.53 mIU/L, close to our 0.50 mIU/L for males. However, the upper FT4 limit in Lebanon was lower than ours at 19.78 pmol/L for females and 18.3 pmol/L for males. Like our study, the Lebanese study observed lower FT3 values in men but did not apply gender partitioning. In Sudan, a narrower TSH range (0.50–3.1 mIU/L) was established without gender partitioning. Unlike our results, females had lower TSH levels than males [26], though no significant gender differences were found in the Darfur region. In Iran, a study in Isfahan [27] compared TSH and T4 RIs in 2006 and 2011, concluding that the recent RI for TSH (0.7–4.9) had higher limits than the manufacturer’s. Another study in Tehran [28] found no significant differences in TPOAb levels by gender or age, similar to our findings, with a maximum value of 18.38 IU/mL compared to our 23.87 IU/mL. Iodine concentration positively correlated with TPOAb rates [29]. In Turkey, RIs for TSH (0.41–4.25 mIU/L), FT4 (7.85–13.64 pmol/L), and FT3 (4.02–5.90 pmol/L) showed no gender differences, unlike our study. The authors also stressed the need for country-specific RIs [30]. It should be noted that these comparisons should be interpreted cautiously, as differences in immunoassay platforms, calibration methods, analytical sensitivities, and laboratory procedures may contribute to variability in reported thyroid hormone reference intervals.

Iodine status is a key factor affecting thyroid function and thyroid biomarker reference intervals across populations [31]. Both iodine deficiency and excess can influence thyroid parameters due to a U-shaped relationship between iodine intake and thyroid disorder risk [32,33]. In iodine-deficient regions, such as parts of the Middle East and Europe, TSH reference intervals are lower. Conversely, countries with sufficient iodine, like the United States, Canada, Japan, and South Korea, exhibit relatively higher TSH intervals [34,35,36,37]. Our study findings are consistent with this pattern and may reflect the improving iodine status in Jordan. Previous reports have shown that Jordan experienced iodine deficiency in the early 1990s, with substantial improvement observed following the implementation of the national universal salt iodization program in 1995, resulting in adequate iodine intake at the population level in subsequent national assessments [19]. Jordan has since maintained mandatory salt iodization and continuous monitoring programs to support adequate iodine intake and reduce iodine deficiency disorders. The population-specific RIs established in this study are critical for improving thyroid disease diagnosis accuracy and avoiding unnecessary treatments that may impose economic burdens and harm public health. Utilizing the new RIs, results show that the prevalence of OHypo is significantly high compared to other countries, including the UK, USA, China, Japan, Australia, and Brazil [38]. It also found hypothyroidism to be more prevalent than hyperthyroidism in Jordan, with both conditions more common in females.

It should also be noted that the overall prevalence estimates reported in the present study reflect the combined burden of previously diagnosed thyroid disorders and newly identified biochemical dysfunction cases detected using the Jordanian-specific RIs established in this study. Previously diagnosed cases were incorporated regardless of treatment status, as the primary objective of the prevalence analysis was to estimate the overall population burden of thyroid dysfunction rather than to distinguish treated, untreated, or newly diagnosed disease. Therefore, the reported prevalence estimates should be interpreted within this epidemiological context. There are some limitations to this study. Being cross-sectional, it could not track changes in thyroid function over time. Additionally, fewer men and participants aged 71–79 were included. As this study focused on a specific Jordanian population, ethnicity may influence the symptoms scoring system, affecting the score threshold at which the TSH upper limit increased radically. Consequently, future studies in diverse populations are needed to enhance the validity and generalizability of the proposed scoring system.

## 5. Conclusions

This study contributes to the body of knowledge by providing a comprehensive framework for developing population-based RIs for thyroid disorders. It begins by adopting a robust filtering method that adds another layer to the traditional exclusion criteria by integrating a superior statistical symptom scoring system to refine the identification of the control group. The proposed symptom scoring system provides a basis for investigating the relationship between thyroid symptoms and TSH levels, which contributes to the understanding and assessment of thyroid disorders. This framework can be generalized and utilized for other biomarkers across diverse populations and disorders, serving as a valuable tool for precise disorder prevalence assessments. Furthermore, our study is the first to establish RIs in Jordan and the first in the MENA region to assess the prevalence of thyroid disorders using population-specific RIs.

## Figures and Tables

**Table 1:** Age and gender percentages in the control group.

Gender	% of the Control Group	Age	% of the Control Group
Male	46.40	18–30	23.62
Female	53.60	31–40	33.71
		41–50	24.76
		51–60	13.71
		61–70	3.24
		71–79	0.95

**Table 2:** Partitions sample size and Jordanian RIs with 90% confidence intervals (CI) versus manufacturer’s RIs of TSH, FT3, FT4, TPOAb, and TRAbs.

Biomarker (Unit)	Sample Size (n)	Jordanian RIs Median (2.5th–97.5th Percentile)	90% Confidence Interval 2.5th Percentile	90% Confidence Interval 97.5th Percentile	Manufacturer’s RIs
TSH (mlU/L) (Male)	245	1.72 (0.50–4.18)	0.42–0.61	3.80–5.25	(0.40–4.50)
TSH (mlU/L) (Female)	283	1.91 (0.57–4.61)	0.42–0.76	4.43–5.48	(0.40–4.50)
FT4 (pmol/L) (Male)	245	15.43 (12.00–21.16)	10.92–13.61	18.26–23.11	(9.00–20.00)
FT4 (pmol/L) (Female)	238	15.30 (12.02–24.69)	10.00–12.85	19.29–26.34	(9.00–20.00)
FT3 (pmol/L) (Male)	245	3.07 (2.28–4.17)	2.25–2.88	3.63–4.56	(3.50–6.50)
FT3 (pmol/L) (Female)	283	3.11 (2.53–3.71)	2.50–2.77	3.60–3.76	(3.50–6.50)
TPOAb (IU/mL)	528	11.49 (0.83–23.87)	0.30–3.38	19.86–25.04	(0–34)
TRAbs (IU/L)	528	0.54 (0.3–1.89)	0.30–0.35	1.42–3.75	(0–0.9)

**Table 3:** Diagnosis of thyroid disorders using the manufacturer RIs versus the Jordanian RIs (females).

	Age	Normal (%)	Overt Hyperthyroidism (%)	Overt Hypothyroidism (%)	Subclinical Hyperthyroidism (%)	Subclinical Hypothyroidism (%)
Manufacturer RIs	18–30	92.06	0.26	2.44	0.61	4.62
	31–40	92.88	0.39	1.95	0.68	4.10
	41–50	91.57	0.46	2.22	0.46	5.28
	51–60	89.95	0.87	3.12	1.04	5.03
	61–70	89.29	0.60	2.98	2.38	4.76
	71–79	90.00	0.00	6.67	0.00	3.33
	Overall	91.84	0.44	2.38	0.69	4.65
Jordanian RIs	18–30	92.65	0.18	1.50	1.24	4.42
	31–40	92.07	0.29	2.25	1.67	3.72
	41–50	91.38	0.28	3.66	1.12	3.56
	51–60	90.42	0.52	3.48	1.74	3.83
	61–70	89.09	0.00	3.64	3.64	3.64
	71–79	93.33	0.00	3.33	0.00	3.33
	Overall	91.84	0.26	2.59	1.45	3.87

**Table 4:** Diagnosis of thyroid disorders using the manufacturer RIs versus the Jordanian RIs (males).

	Age	Normal (%)	Overt Hyperthyroidism (%)	Overt Hypothyroidism (%)	Subclinical Hyperthyroidism (%)	Subclinical Hypothyroidism (%)
Manufacturer RIs	18–30	92.86	0.82	2.75	0.27	3.30
	31–40	95.01	0.00	1.94	0.28	2.77
	41–50	94.85	0.47	0.94	0.70	3.04
	51–60	94.36	0.31	1.88	0.94	2.51
	61–70	93.18	0.57	1.14	1.14	3.98
	71–79	93.55	0.00	3.23	0.00	3.23
	Overall	93.82	0.48	1.77	0.75	3.17
Jordanian RIs	18–30	92.63	0.85	1.98	1.13	3.40
	31–40	94.66	0.00	1.12	0.56	3.65
	41–50	94.09	0.47	1.65	1.42	2.36
	51–60	93.67	0.63	3.80	1.27	0.63
	61–70	90.86	0.00	1.71	4.00	3.43
	71–79	91.80	0.00	4.92	1.64	1.64
	Overall	92.97	0.49	2.12	1.69	2.72

**Table 5:** Counts and percentages of participants who were over-diagnosed or under-diagnosed.

		Thyroid Patients Reclassified as Normal Participants (Count, %)		Subclinical Hypothyroidism Reclassified as Overt Hypothyroidism (Count, %)		Subclinical Hyperthyroidism Reclassified as Overt Hyperthyroidism (Count, %)	
	Age	Count	%	Count	%	Count	%
Over diagnosis	18–30	2	0.13	20	1.33	1	0.07
	31–40	0	0.00	14	1.01	1	0.07
	41–50	2	0.13	14	0.94	2	0.13
	51–60	4	0.45	8	0.90	2	0.22
	61–70	0	0.00	3	0.88	2	0.59
	71–79	1	1.10	1	1.10	0	0.00
	Overall	9	0.16	60	1.05	8	0.14
		**Normal Reclassified As Thyroid Patients’ Participants (Count, %)**		**Subclinical Hypothyroidism Reclassified as** **Overt Hypothyroidism (Count, %)**		**Subclinical Hyperthyroidism Reclassified as** **Overt Hyperthyroidism (Count, %)**	
	**Age**	**Count**	**%**	**Count**	**%**	**Count**	**%**
Under diagnosis	18–30	21	1.40	10	0.67	0	0.00
	31–40	18	1.30	14	1.01	0	0.00
	41–50	22	1.48	32	2.15	0	0.00
	51–60	9	1.01	18	2.02	1	0.11
	61–70	8	2.35	5	1.47	0	0.00
	71–79	2	2.20	1	1.10	0	0.00
	Overall	80	1.41	80	1.41	1	0.02

## Data Availability

Some or all datasets generated and analyzed during the current study are not publicly available due to privacy and ethical restrictions but are available from the corresponding author upon reasonable request.
